# FundusNet: A Deep-Learning Approach for Fast Diagnosis of Neurodegenerative and Eye Diseases Using Fundus Images

**DOI:** 10.3390/bioengineering12010057

**Published:** 2025-01-13

**Authors:** Wenxing Hu, Kejie Li, Jake Gagnon, Ye Wang, Talia Raney, Jeron Chen, Yirui Chen, Yoko Okunuki, Will Chen, Baohong Zhang

**Affiliations:** Research Department, Biogen, Inc., 225 Binney St., Cambridge, MA 02142, USA; wenxing.hu@biogen.com (W.H.); jake.gagnon@biogen.com (J.G.);

**Keywords:** fundus, neurodegenerative disease, vision transformer

## Abstract

Early-stage detection of neurodegenerative diseases is crucial for effective clinical treatment. However, current diagnostic methods are expensive and time-consuming. In this study, we present FundusNet, a deep-learning model trained on fundus images, for rapid and cost-effective diagnosis of neurodegenerative diseases. FundusNet achieved superior performance in age prediction (MAE 2.55 year), gender classification (AUC 0.98), and neurodegenerative disease diagnosis—Parkinson’s disease AUC 0.75 ± 0.03, multiple sclerosis AUC 0.77 ± 0.02. Grad-CAM was used to identify which part of the image contributes to diagnosis. Imaging biomarker interpretation demonstrated that FundusNet effectively identifies clinical retina structures associated with diseases. Additionally, the model’s high accuracy in predicting genetic risk suggests that its performance could be further enhanced with larger training datasets.

## 1. Introduction

Neurodegenerative diseases (NDD) affect approximately 15% of the worldwide population [1]. Most NDDs cannot be cured so far, and current medications can only slow down the development. As a result, early-stage diagnosis, which requires more frequent screening, is crucial for effective treatment. However, traditional diagnostic methods rely on costly imaging scans or time-intensive evaluations by neurologists, posing a significant financial and logistical burden for both patients and insurance providers when performed frequently.

The retina, often considered an extension of the brain, is the only part of the central nervous system that can be visualized non-invasively. Abnormal changes in optical nerves may indicate pathological progression [2]. Optical scans, such as fundus photography, can detect structural abnormalities in the retina, providing a low-cost method to detect structural abnormalities in the retina, enabling early diagnosis of neurodegenerative diseases. Applying artificial intelligence models to the reading of fundus images can further reduce the cost of ophthalmologists and accelerate patient prescreening. It has been demonstrated that fundus images can predict age, gender, smoking status, and other cardiovascular factors [3]. Moreover, fundus imaging is widely used to diagnose eye diseases. For instance, glaucoma, a group of eye disorders that cause vision loss due to optic nerve damage, can be accurately detected using deep-learning models, which have matched the diagnostic performance of ophthalmology specialists [4,5]. These models are also effective in segmenting and quantifying image-based biomarkers. Another example is age-related macular degeneration (AMD), a chronic eye disease that leads to gradual vision loss in the center of the visual field. Similarly, deep-learning models have demonstrated superior performance compared to retinal specialists in patient classification and in detecting large drusen and pigmentary abnormalities [6].

In this study, we focus on diagnosing neurodegenerative diseases such as multiple sclerosis (MS) and Parkinson’s disease (PD). Both MS and PD are primarily neurodegenerative diseases rather than eye diseases. Their diagnosis is more costly, especially MS. The clinical diagnosis of MS needs information on Magnetic Resonance Imaging (MRI) or cerebrospinal fluid (CSF). Parkinson’s disease is diagnosed based on a patient’s clinical features or symptoms, as there is no specific test for the disease. This motivates us to develop a deep-learning model that uses fundus images for rapid and low-cost MS and PD diagnosis.

For MS diagnosis, several studies [7,8] used optical coherence tomography (OCT), which provides more detailed retinal structural information but is more expensive than fundus scan. Despite this, the diagnosis results were low, with AUCs ranging from 0.66 to 0.75. Moreover, processing OCT images is slower because it involves segmenting various retinal layers and structures. Developing a better deep-learning model using fundus images could achieve better results while being faster and more cost-effective.

Explainability is critical for clinical adoption, as interpretable models enable ophthalmologists to better understand the decisions made by deep-learning models. Heatmap-based visualizations like Grad-CAM [9] have been widely used to highlight disease-relevant regions in fundus images. In our work, We utilized saliency visualization methods to enhance our model explainability. The results demonstrated that our model effectively identifies abnormal structures associated with diseases, providing valuable insights into its diagnostic decisions.

The rest of the paper is organized as follows. Section 2 describes the dataset and computational model. Section 3 presents the experimental result of using fundus for disease diagnosis and the prediction of age and gender. Section 4 concludes the work.

## 2. Materials and Methods

Fundus images from the UK Biobank (UKBB) data were used in our work. FundusNet employs multiple convolutional neural network (CNN) models and vision transformer (ViT) models. All models were initialized using default weights, which were well-trained on super large-scale image datasets and then fine-tuned on the UKBB fundus images. After that, to further enhance classification and regression accuracy, we used ensemble learning to combine their results via a bagging approach.

### 2.1. UKBB Dataset

The UK Biobank [10,11,12] is a population-scale database that contains extensive medical, genetic, and imaging information from half a million participants across the UK. Launched in 2006, the UKBB aims to improve the prevention, diagnosis, and treatment of a wide range of illnesses by providing researchers with unprecedented access to a vast array of high-quality data. A total of 80,000 of the half million participants have contributed genetic information, fundus images of the retina, and medical records of a wide range of diseases, coded as the ICD10 score, through questionnaires, physical measurements, and biological samples.

**Figure 1:**
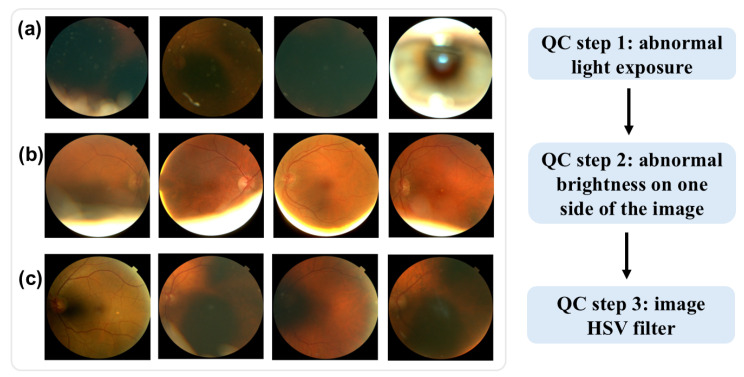
Examples of low-quality fundus images. (**a**) abnormal light exposure. (**b**) over-brightness at one side of the image. (**c**) HSV quality control.

Low-quality images were excluded through a three-step quality control (QC) process for the fundus images, as shown in Figure 1. First, images with abnormal light exposure during optical scan were excluded. As shown in Figure 1a, these images are either excessively dark or overly bright, offering minimal information about the retina’s structure. Second, images with over-brightness at one side were excluded, as shown in Figure 1b. Lastly, we applied HSV (hue, saturation, and value) thresholds (hue < 15 or >100) to filter out additional low-quality images. This HSV-based QC step, which has been employed in other studies [13,14] for fundus image QC, further eliminated images with limited retinal structure detail, as shown in Figure 1c.

### 2.2. Convolutional Neural Networks

Several convolutional neural networks (CNN) were used in this work. This section provides a brief description of those CNN models.

RegNet [15], proposed by FAIR in 2020, can systematically control the network’s width, depth, and group width through a simple yet powerful design space, enabling an optimal balance between performance and computational complexity. There are two variants of RegNet: RegNetX and RegNetY. RegNetX is simpler and more efficient, while RegNetY is more powerful using Squeeze-and-Excitation modules which can capture feature interdependencies better. EfficientNet [16] introduces a new scaling method that uniformly scales all dimensions of depth, width, and resolution using a compound coefficient. This innovative approach allows EfficientNet to achieve superior performance and efficiency compared to previous CNN architectures. NFnet [17] modifies ResNet architecture using alternative ways to reduce gradient exploding. Without batch normalization, the network converges faster and improves robustness to various hyperparameter settings and different training scenarios. The new methods introduced by NFnet include Scaled Weight Standardization and Adaptive Gradient Clipping, which stabilize the gradients and control the gradient norms to avoid the gradient exploding problem.

### 2.3. Vision Transformer Models

Besides convolutional neural networks, several vision transformer models were also used in our work. Vision transformer (ViT) [18] replaces the conventional hierarchical feature extraction of CNNs with a self-attention mechanism, which treats images as sequences of patches. This allows ViT to capture global dependencies and relationships across different parts of the image, effectively learning contextual information crucial for accurate visual recognition. ViT achieves superior results on various image classification benchmarks. Data-efficient image Transformers (DEiT) [19] introduce a teacher-student technique in which a student network learns from a teacher network through attention based on a distillation token. This enables faster adoption of large, well-trained models on smaller datasets.

Bidirectional Encoder representation from image Transformers (BEiT) [20] is a self-supervised vision transformer model. BEiT surpasses DEiT according to benchmarking on ImageNet-1K. Class-Attention in Image Transformers (CAiT) [21] is another variant of ViT, which conducts class embedding at a later layer and freezes the patch embeddings afterward. This improves ViT’s performance closer to that of the SOTA convolutional networks. VOLO [22] improves ViT’s performance by introducing outlook attention, which learns intermediate finer-level patches more efficiently into tokens.

**Figure 2:**
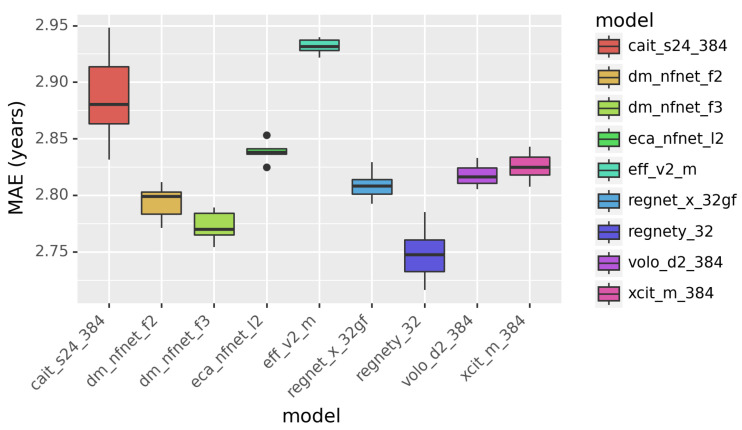
The performance of individual models in predicting age. Mean absolute error (MAE) is in years.

Both the CNN-based models and the ViT-based models were implemented using PyTorch Image Models (https://github.com/huggingface/pytorch-image-models?tab=readme-ov-file, last accessed on 20 December 2024). Pretrained weights were pretrained https://github.com/huggingface/pytorch-image-models?tab=readme-ov-file#models, last accessed on 20 December 2024 based on ImageNet. All experiments were conducted on a Tesla V100-SXM2-32GB GPU. Note that each model has multiple variants due to the different settings of hyper-parameters. For example, ResNet-50 and ResNet-152 are two variants of ResNet that differ in the number of layers (50 and 152 layers, respectively). Variants with larger sizes, e.g., a larger number of layers or parameters, can achieve better performance but require more GPU memory and longer training time. Limited by both time and hardware, we selected a proper variant for each model. As a result, the training time and memory cost of different models are comparable. This was accomplished by selecting appropriately sized submodels. For instance, RegNet offers various submodels such as RegNet8g (8G parameters), RegNet16g (16G parameters), and RegNet32g (32G parameters). To have comparable training time and memory cost for each model, We selected a proper-sized submodel. After that, we still trained the model using standard optimization and de-overfitting techniques to ensure the final networks were optimal. The names of the final selected subnetworks were presented in Figure 2. The submodels, along with their pretrained weights and the detailed architecture specifications, are available at https://github.com/huggingface/pytorch-image-models?tab=readme-ov-file#models, last accessed on 20 December 2024.

To address the overfitting problem, we used dropout on the last fully connected layers to randomly mask/prune nodes and image augmentation to increase the sample size and variance. Image augmentation includes random rotation, random cropping, etc. In addition, we used a learning rate scheduler that manages the decay of the learning rate to accelerate convergence.

Raw rectangular fundus images were squarely cropped and then resized to 384 by 384 pixels. CNN-based models are flexible with respect to the size of input images, while ViT-based models require a fixed input size of 384 pixels. As the image augmentation step includes random cropping which reduces the size to less than 384 pixels, a margin patch was added to each image before feeding into the ViT-based models.

### 2.4. Model Ensembling

Ensemble learning is a method that builds a stronger classifier by combining multiple relatively weaker classifiers by either bagging or boosting. The *Error*-ambiguity decomposition equation below elucidates the relationship between the ensemble model and the individual classifiers.

$$Error_{ensemble}=Error_{IndividualClassifier}-Diversity_{classifiers}$$

**Figure 3:**
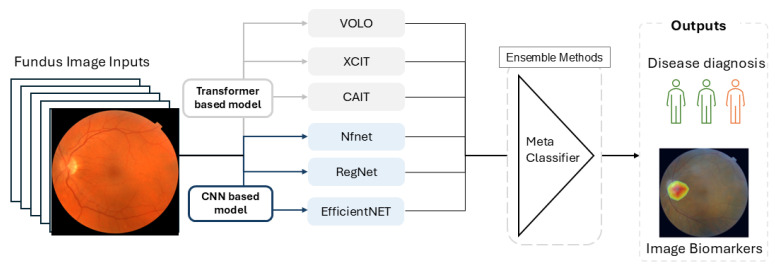
The workflow of FundusNet model.

According to the *Error*-ambiguity equation, there are two ways to minimize the error of the ensemble model: increasing the accuracy of individual classifiers and enhancing the between-classifier diversity. To enhance diversity among classifiers, we utilized multiple CNN and ViT models, each trained 10 times using bootstrapped resampling of 80% of the training data. The outputs were then passed to an ensemble classifier, which combines results through majority voting for classification or by averaging values for prediction. The workflow of FundusNet is illustrated in Figure 3.

### 2.5. Result Interpretation and Image Biomarker Identification

Beyond predicting phenotypes and disease diagnosis, interpreting the results is also important for building trust in end users such as doctors and patients and for extracting key image biomarkers for research. Feature-map-based interpretation methods have been proposed to explain the results of convolutional neural networks. Class activation maps (CAM) method [23] can generate pixel-level activation maps indicating the contribution of each pixel by combining intermediate feature maps, i.e., convolutional layers. Due to the recalculation of weights for each image, running CAM is slow, and consequently, its application is limited. To address this, the Gradient-weighted CAM (Grad-CAM) method [9] and Grad-CAM++ [24] improved CAM by computing weights based on existing gradient information, which dramatically reduces the time cost for result interpretation.

When interpreting image classification results using Grad-CAM-based methods, a specific layer of a deep-learning model is required rather than an ensemble of multiple models. In our experiments, the final convolutional layer of the best-performing model, i.e., RegNetY_32, is used for Grad-CAM.

## 3. Results

The FundusNet model was used on fundus images from UKBB to predict age, gender, and neurodegenerative diseases, including multiple sclerosis (MS), Parkinson’s disease (PD), glaucoma, and age-related macular degeneration (AMD). The mean absolute error (MAE) is used as a metric for evaluating continuous variable prediction, i.e., age, and the area under the receiver operating characteristic curve (AUC) is used as a metric for evaluating classification results.

### 3.1. The Performance of Individual Models

We compared different CNN and ViT models for age prediction. The results are presented in Figure 2. The performance is quantified in MAE in years. Smaller MAE indicates better performance. According to Figure 2, RegNetY outperforms other models, including the ViT-based models. This demonstrates that Vision Transformers are less powerful than the CNN-based models in image classification tasks.

### 3.2. The Prediction of Age and Gender

Age and gender are considered phenotype traits. Using fundus images to predict age and gender demonstrates that fundus images can predict phenotypes and diseases. We used FundusNet, shown in Figure 3, for age prediction and gender prediction. Experiments were conducted using the UKBB dataset, with 167,000 images. We compared the results to that of previous research [3]. The study [3] used a larger dataset, with 611 k images, including two datasets—the UKBB dataset and the EyePACS dataset.

As demonstrated in Table 1, despite using a much smaller dataset (the 167 k images are a subset of the 611 k images used in [3]), our proposed work, FundusNet, achieved superior performance in both age prediction and gender classification compared to the result of Google’s team [3]. Please note that the improvement could result from the different age distributions of the training dataset. The difference between real age and predicted age can be used as a disease indicator/biomarker, demonstrated in paper [25]. A larger gap between real age and predicted age may suggest abnormal aging of the retina or a certain retina/neurodegenerative disease.

### 3.3. Classification of Neurodegenerative Diseases

Table 2 presents the diagnosis results of neurodegenerative and eye diseases using FundusNet. The sample size column describes how many diseased samples are available. For each disease, we selected the same number of healthy control samples with gender and age-matched to the disease group. As a result, all the four rows in Table 2 are balanced classification.

Both AMD and glaucoma are conventional eye diseases, and fundus images are used for clinical diagnosis.

For AMD, a previous AI work [6] achieved an AUC of 0.9, comparable to the diagnosis of retina specialists, which is higher than our AUC result of 0.75. However, the AMD training data in our work, i.e., UKBB with 219 AMD patients, is much smaller than the AMD data (59,302 images) used in paper [6]. The small training data limited our performance.

For glaucoma diagnosis, a previous AI work [5] achieved an AUC of 0.81 on the Singapore Indian Eye Study (SINDI) dataset, which included 5783 samples. Our result (on 1023 UKBB glaucoma samples), with an AUC of 0.82, shows a slight improvement.

Both MS and PD are more neurodegenerative diseases rather than eye diseases. Their diagnosis is more costly, making them the central focus of our work, especially MS. The clinical diagnosis of MS needs information on Magnetic Resonance Imaging (MRI) or cerebrospinal fluid (CSF). Parkinson’s disease is diagnosed based on a patient’s clinical features or symptoms, as there is no specific test for the disease. As a result, FundusNet-aided rapid and low-cost MS and PD diagnosis is the primary motivation and contribution of our work.

Our work achieved an AUC of 0.75 for PD diagnosis, slightly better than previous work [26] that reported AUCs ranging from 0.56 to 0.77 using deep neural networks on UKBB data.

For MS diagnosis, our work achieved an AUC of 0.77, which outperformed previous works [7] (AUC of 0.66–0.73), [8] (AUC of 0.75). This work [7] used optical coherence tomography (OCT), which provides more detailed retinal structural information and is more expensive than a fundus scan. Processing OCT images is slower, as it requires segmentation of various retinal layers and structures. Our work achieved better results and is faster and less costly.

### 3.4. Result Interpretation and Image Biomarkers of Disease Diagnosis

The Grad-CAM method was used to interpret FundusNet’s results in disease diagnosis. As introduced in the Method section, Grad-CAM can generate pixel-wise activation maps that highlight key image biomarkers with the highest contribution to disease diagnosis. Identifying correct image biomarkers is important for end users such as doctors and patients and is also beneficial for future research on the same disease. The Grad-CAM method is fast due to its algorithmic design, making it feasible for real-time interpretation.

Grad-CAM requires a convolutional layer for result interpretation. We used the last convolutional layer of the most accurate model (see Section 3.1), i.e., RegNetY, for interpreting results when running Grad-CAM to generate saliency/activation maps.

**Figure 4:**
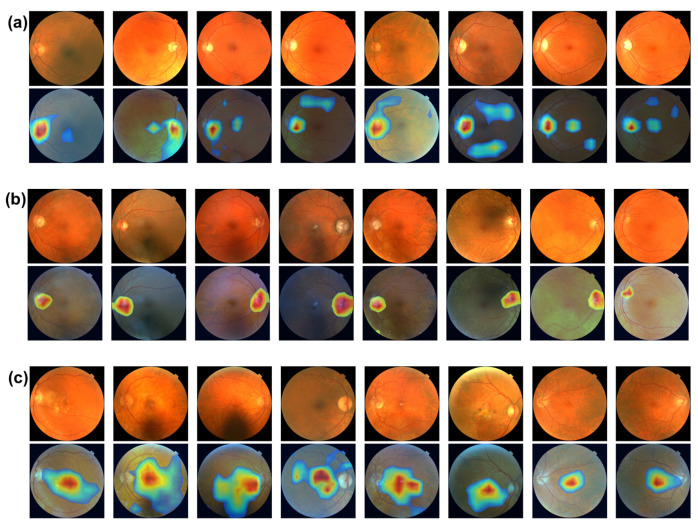
(**a**) Image biomarkers for MS. (**b**) Image biomarker for glaucoma. (**c**) Image biomarkers for AMD.

Figure 4 shows the image biomarker interpretations for MS, glaucoma, and AMD, respectively. From Figure 4a,b, optic disc, the brighter circular area on fundus images is highlighted as image biomarkers for both MS and glaucoma disease. The optic disc, also known as optic nerve head, is where the optic nerves connect to the retina. It is the point where the ganglion cell axons (nerve fibers) exit the eye to form the optic nerve, which transmits visual information from the retina to the brain. The optic disc is important in clinical diagnosis. Changes in its appearance can indicate various eye conditions, such as glaucoma and optic neuritis (a common eye problem that affects MS patients). Increased pressure within the eye can cause cupping of the optic disk, a key indicator in diagnosing glaucoma. Figure 4c shows that the macula, the darker small central area of the retina, is identified as an image biomarker for AMD. The macula is responsible for high acuity vision, which is essential for eye function. Located near the optic disk, the macula contains a high density of photoreceptor cells, particularly cones, which are responsible for color vision and detailed central vision. The central part of the macula, called the fovea, is the most sensitive and is crucial for sharp central vision. Macular degeneration is a leading cause of vision loss among people aged 50 years and older.

### 3.5. Predicting Polygenic Risk Score

The prediction of neurodegenerative diseases is limited by the small sample size of patients, as the UKBB is population-scale data rather than a cohort focused on a specific disease. This limited the performance of FundusNet as deep-learning models require a large number of samples. To increase sample size, we calculated the Polygenic Risk Score (PRS) of MS disease [27] and used participants with higher MS PRS as the high MS risk group. PRS is calculated based on the cumulative effect of numerous genetic variants across the genome, each contributing a small amount to the overall risk of developing complex traits or conditions.

**Figure 5:**
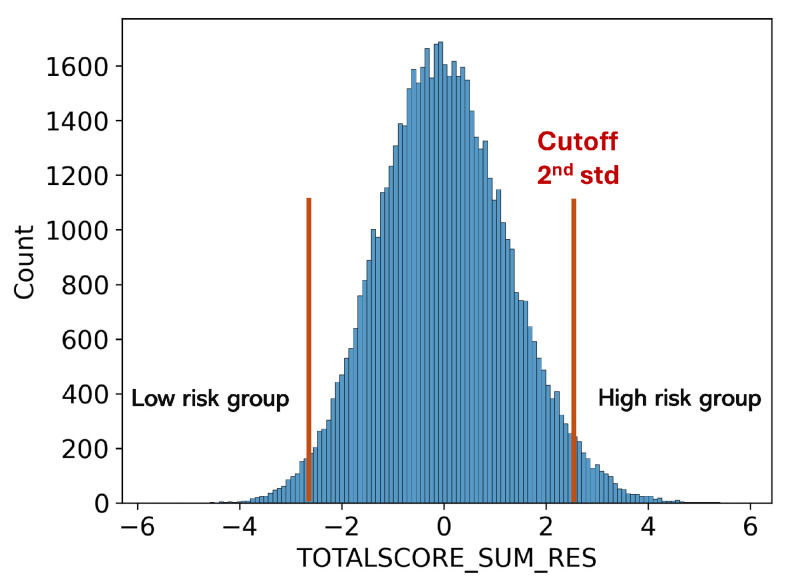
Low and high MS risk groups defined using PRS score.

As shown in Figure 5, participants with a higher PRS (above mean +2std or 3std) are used as the high-risk MS group, while those with a lower PRS (below mean −2std or 3std) are used as the low-risk MS group. We used the same FundusNet model with fundus images to classify the high and low MS PRS groups. When using FundusNet to classify the two groups, the most extreme scenario achieved 0.999 AUC, as shown in Table 3, demonstrating that fundus images are highly discriminative in predicting neurodegenerative disease-related genetic variants. Moreover, this may suggest that high accuracy in predicting genetic risk suggests that the proposed FundusNet’s performance could be further enhanced with larger training datasets.

## 4. Conclusions

In this study, we present FundusNet, a model designed to use fundus images for diagnosing neurodegenerative diseases (NDD). Our model achieved slightly better results for gender and age prediction. The improvement could result from the different age distributions of the training datasets. For NDD diagnoses, such as multiple sclerosis (MS) and Parkinson’s disease (PD), FundusNet achieved better results than previous studies, though performance was still limited by the small sample size of patients with these conditions. Imaging biomarker analysis showed that FundusNet effectively identified retina structures relevant to disease diagnosis. Additionally, the model’s strong performance in predicting genetic risk scores underscores fundus images’ potential for diagnosing diseases as larger datasets become available. These results demonstrate that FundusNet could be used as a fast and cost-effective tool for neurodegenerative disease diagnosis.

It is possible that some other factors could contribute to the high AUC in PRS results, which could be a limitation of the results. However, the data size in the PRS experiment is large enough (5–10 k samples), and the PRS classification results are extremely high (AUC of 0.95–0.99). Moreover, the high and low-risk groups are of a balanced size. Therefore, this limitation does not affect our conclusion that when datasets of larger sample sizes are available, disease diagnosis using fundus images could become more accurate, which is the primary motivation of the PRS experiment.

## 5. Code Availability

The source code, local installation guide and complete tutorial of visualization and analysis tool are provided at https://github.com/interactivereport/FundusNet, last accessed on 20 December 2024. With broad adoption and contribution in mind, FundusNet is released under the MIT License.

## Figures and Tables

**Table 1:** Result: prediction of age and gender.

Phenotype	Google’s Paper [3] (611 k Images)	FundusNet (167 k Images)
Gender (AUC, higher is better)	0.97	0.98
Age (MAE, lower is better)	3.26	2.55

**Table 2:** Result of neurodegenerative and eye diseases diagnosis.

Disease	Sample Size	AUC (mean±std)
AMD	219	0.75±0.04
PD	263	0.75±0.03
MS	349	0.77±0.02
Glaucoma	1023	0.82±0.01

**Table 3:** Result of MS PRS risk groups classification using fundus images.

MS PRS	Sample Size	AUC
2nd standard derivation	10,743	0.953
3rd standard derivation	5549	0.999

## Data Availability

The UKBB fundus images and genetic data used in this paper are publicly available at https://www.ukbiobank.ac.uk/, last accessed on 20 December 2024.

## Abbreviations

The following abbreviations are used in this manuscript:

SOTA	state-of-the-art
CNN	convolutional neural networks
ViT	Vision Transformer
BEiT	Bidirectional Encoder representation from image Transformers
DEiT	Data-efficient image Transformers
CAiT	Class-Attention in Image Transformers
PRS	Polygenic Risk Score
MAE	mean absolute error
CAM	Class activation maps
Grad-CAM	Gradient-weighted CAM
HSV	hue, saturation, and value
QC	quality control
UKBB	UK BioBank
NDD	neurodegenerative diseases
AMD	age-related macular degeneration
MS	multiple sclerosis
AD	Alzheimer’s disease
PD	Parkinson’s disease
